# Tenecteplase for ST-elevation myocardial infarction in a patient treated with drotrecogin alfa (activated) for severe sepsis: a case report

**DOI:** 10.1186/1752-1947-3-109

**Published:** 2009-11-05

**Authors:** Lillian Barra, Jeffrey Shum, J Geoffrey Pickering, Raymond Kao

**Affiliations:** 1 Division of Critical Care, Department of Medicine, University of Western Ontario, Commissioner's Rd, London, Ontario, Canada; 2 Division of Cardiology, Department of Medicine, University of Western Ontario, Commissioner's Rd, London, Ontario, Canada

## Abstract

**Introduction:**

Drotrecogin alfa (activated) (DrotAA), an activated protein C, promotes fibrinolysis in patients with severe sepsis. There are no reported cases or studies that address the diagnosis and treatment of myocardial infarction in septic patients treated with DrotAA.

**Case presentation:**

A 59-year-old Caucasian man with septic shock secondary to community-acquired pneumonia treated with DrotAA, subsequently developed an ST-elevation myocardial infarction 12 hours after starting DrotAA. DrotAA was stopped and the patient was given tenecteplase thrombolysis resulting in complete resolution of ST-elevation and no adverse bleeding events. DrotAA was restarted to complete the 96-hour course. The sepsis resolved and the patient was discharged from hospital.

**Conclusion:**

In patients with severe sepsis or septic shock complicated by myocardial infarction, it is difficult to determine if the myocardial infarction is an isolated event or caused by the sepsis process. The efficacy and safety of tenecteplase thrombolysis in septic patients treated with DrotAA need further study.

## Introduction

Sepsis with multi-organ failure has a high incidence and a mortality rate of 30-50% [1]. The pathophysiology of sepsis is an inflammatory and procoagulant state triggered by infection. Activated protein C (APC) plays an important role by promoting fibrinolysis and inhibiting thrombosis and inflammation. The PROWESS trial reported a 6.1% absolute reduction in 28-day mortality for septic patients treated with drotrecogin alfa (activated) (DrotAA) [2]. Subsequent studies and subanalyses have suggested that most of the benefit is seen in patients with high illness severity scores or multiple organ dysfunction [3]. In our center, patients are treated with DrotAA (24 mcg/kg/hour × 96 hours) when there is evidence of infection, signs of systemic inflammatory response syndrome plus two or more organ system failures or one organ system failure and APACHE II score ≥25. Patients are excluded if death is perceived to be imminent, sepsis-induced organ failure has lasted more than 48 hours or there is an increased risk of life-threatening or intracranial bleeding.

## Case presentation

A 59-year-old Caucasian man presented to the emergency department after a motor vehicle collision and was found to have a right lower lobe pneumonia but no other injuries. He was discharged home on azithromycin. He had a history of type 2 diabetes, asthma, hypertension and hyperlipidemia, but he was a non-smoker with a negative history for coronary heart disease or strokes. His medication included ventolin, glyburide, metformin, quinipril, atorvastatin and aspirin. Two days later, he presented to the same emergency department with a decreased level of consciousness and respiratory distress, requiring mechanical ventilation and transfer to the intensive care unit (ICU).

On admission, he was hemodynamically stable and his temperature was 39.2°C. His white blood cell count was 11.3 × 10^9^/L, hemoglobin 131 g/L and platelets 150 × 10^9^/L. Arterial blood gas showed a PaO_2 _97 mmHg on 100% oxygen, PaCO_2 _54 mmHg, bicarbonate 25 mmol/L and pH 7.32. His lactate level was 2.1 mmol/L, SvO_2 _76% and troponin I was elevated at 0.6 μg/L. His international normalized ratio (INR), partial thromboplastin time (PTT), liver enzymes and electrolytes were normal, but creatinine was elevated at 211 μmmol/L. His chest X-ray demonstrated worsening of pneumonia and his electrocardiogram (ECG) showed no evidence of ischemia. Intravenous antibiotics (cefotaxime) were given pending microbiological culture results.

Four hours after presentation, his mean arterial pressure (MAP) decreased from 77 mmHg to 60 mmHg and he was unresponsive to fluid resuscitation alone. There were no ischemic changes on ECG monitoring and further troponin I testing was not performed. The rest of the laboratory tests were unchanged. It was felt that the patient had developed severe sepsis secondary to community-acquired pneumonia and norepinephrine plus vasopressin (0.4 U/minute) were initiated for blood pressure support. His APACHE score was 26, and with two dysfunctional organs, DrotAA and hydrocortisone were initiated as part of severe sepsis treatment.

**Figure 1:**
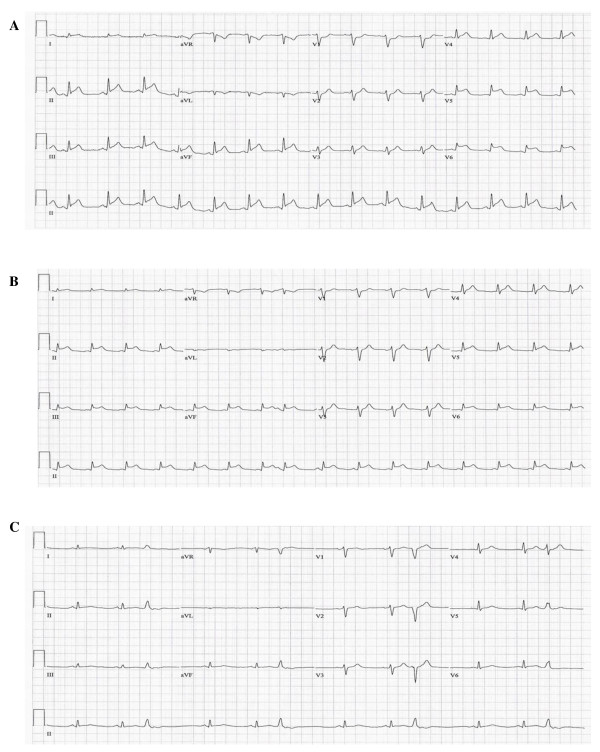
**Electrocardiogram response to tenecteplase lysis**. (A) Electrocardiogram before tenecteplase thrombolysis; 2 mm ST-elevations in leads in I, II, III, aVF and 3 mm ST-elevations in V5, V6. (B) Two hours post-tenecteplase; 50% decrease in ST-elevations. (C) Twenty-four hours post-tenecteplase; complete resolution of ST-elevations.

Twelve hours after starting DrotAA, the patient was noted to have ST-elevations on the cardiac monitor and the ECG demonstrated 2-3 mm ST-segment elevations in the inferolateral leads (Figure 1A). The troponin I level was elevated to 98.89 μg/L. Immediate primary percutaneous coronary intervention was not available at the hospital, so tenecteplase (TNK; 40 mg intravenous bolus) was administered followed by intravenous heparin titrated by a nomogram to maintain a PTT of 60-80 seconds for a total of 4 hours. The DrotAA infusion was stopped 4 hours before initiating TNK because of the theoretical risk of increased bleeding by combining it with TNK. Similarly, other than aspirin, no other antiplatelet agents were used. There was acceptable resolution of the ST-segment elevation by 1.5 hours post TNK administration (Figure 1B, C). The DrotAA infusion was restarted 8 hours post TNK treatment for a total of 96 hours. An echocardiogram obtained 24 hours after the event demonstrated severe global hypokinesis of the left ventricle with an ejection fraction (EF) of 20-25% and reduced right ventricular function.

**Figure 2:**
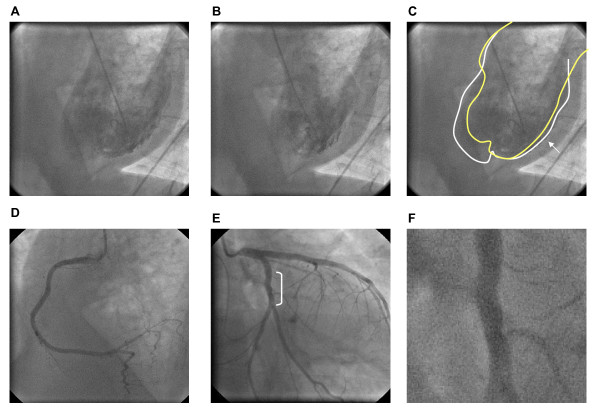
**Ventriculogram and coronary angiogram day 9 post ST-elevation myocardial infarction**. (A, B) Ventriculogram (left anterior oblique view) in diastole (A) and systole (B). (C) Tracings of ventricular wall in diastole (white) and systole (yellow); minimal contraction of posterolateral wall (arrow) indicative of akinesis. (D) Left anterior oblique view of right coronary artery with insignificant irregularities. (E) Right anterior oblique view of left coronary system; normal left anterior descending artery, irregularities of left circumflex artery shown in detail in (F).

In the 24 hours following TNK, the patient's blood pressure improved and he no longer required norepinephrine and vasopressin. His blood cultures grew *Streptococcus pneumoniae *sensitive to cefotaxime and azithromycin. On day 5 of admission, the patient was extubated and transferred out of the ICU. Cardiac catheterization was performed (Figure 2) and demonstrated insignificant irregularities of the right coronary artery and a normal left anterior descending artery. There was an irregularity in the left circumflex artery that could have been the remnants of the culprit lesion. The EF was 45% with akinesis of a segment of the posterolateral and inferior wall. The patient was subsequently discharged home on day 12 after admission.

## Discussion

Myocardial dysfunction is a common complication in patients with severe sepsis with approximately 50% of patients having impairment of ventricular systolic function. The mechanism for the dysfunction is via incompletely characterized depressant mediators [4]. The relationship between elevated cardiac troponin levels and a diagnosis of myocardial infarction due to thrombosis is uncertain in the ICU setting. Ammann *et al*. [5] revealed that more than 70% of ICU patients with elevated cardiac troponin levels did not have flow-limiting coronary artery disease as indicated by stress echocardiography or by findings at autopsy.

The significance of the ST-segment elevation in patients with sepsis is also questioned. Several published reports indicate that acute ST-segment elevations can occur in sepsis with non-significant coronary artery disease [6]. Therefore the ECG changes should be considered in relation to the clinical data at presentation, rather than interpreted as a single diagnostic finding. Assessment for symptoms of ischemia in intubated patients is limited, but recognizing myocardial infarction in these patients is important as it may contribute to increased morbidity and mortality [7]. Certain medications used uniquely in ICU settings may also contribute to ischemic events. Holmes *et al*. [8] found an increase in cardiac arrests in ICU patients on doses of vasopressin greater than 0.05 U/minute.

APC inhibits coagulation by inactivating factor Va and VIIIa and promotes fibrinolysis by inhibition of type 1 plasminogen activator inhibitor (PAI-1). Animal models suggest that APC enhances thrombolysis and prevents re-occlusion in coronary artery thrombosis [9,10]. A small randomized controlled trial investigating the addition of DrotAA versus unfractionated intravenous heparin with tissue plasminogen activator in patients with ST-elevation myocardial infarction (STEMI) found that the DrotAA group had lower levels of PAI-1. The authors concluded that DrotAA may be beneficial in the treatment of acute myocardial infarction, however the study lacked clinical outcomes and the numbers were too small to make any meaningful conclusion regarding the use of DrotAA in STEMI or for prevention of thrombotic events [11].

To the best of our knowledge, our patient is the first reported case of severe sepsis treated with TNK while on DrotAA for STEMI. The differential diagnosis includes streptococcal myocarditis and stress-induced Takotsubo cardiomyopathy with clinical presentations indistinguishable from myocardial infarction. For patients with myocarditis, a definitive diagnosis cannot be made without tissue biopsy and cultures. Takotsubo cardiomyopathy will typically have ST-elevation in the precordial leads, mild to modest elevations in cardiac troponins, hypokinesis of the mid to apical segments of the left ventricle and no critical lesions on cardiac angiogram [12]. Takotsubo has been described with *Streptococcus pneumoniae *infections, as seen in this patient [13]. However, our patient had an inferolateral distribution of ischemia with a marked elevation in troponin I and global hypokinesis, which is atypical for Takotsubo cardiomyopathy.

Our patient with severe sepsis was found to have ST-elevations on ECG, a large troponin rise and global cardiac hypokinesis. Given that our center did not have access to urgent cardiac catheterization, we elected to treat him as a STEMI patient with the accepted standard of TNK thrombolysis followed by heparin infusion. DrotAA was stopped because of the increased risk of bleeding and then resumed for ongoing sepsis 8 hours after the thrombolysis. The patient had resolution of ST-elevations post-thrombolysis with improvement in cardiac function as well as resolution of sepsis with no adverse bleeding events.

A cardiac angiogram performed post-thrombolysis revealed mild irregularities and no coronary artery occlusion, suggesting a non-thrombotic cause for his cardiac event. However, the findings could also reflect successful thrombolysis. This is supported by evidence of posterolateral and inferior wall hypokinesis with left circumflex artery irregularity, corresponding to the initial ECG ST-elevation territory. The global hypokinesis seen on echocardiogram before cardiac catheterization may have been due to a combination of sepsis-induced myocardial dysfunction and a possible ischemic event. Unfortunately, without primary cardiac catheterization, we cannot definitively know whether our patient's cardiac dysfunction was secondary to a thrombotic mechanism versus induced by sepsis.

## Conclusion

Further research is needed on the prognostic significance of elevated cardiac troponins and ST-elevation as well as their relationship to myocardial infarction in critically ill patients. A guideline is also needed to advise treatment strategies in septic patients who develop STEMI/non-STEMI while being treated with other fibrinolytic agents not approved for myocardial infarction such as DrotAA.

## Abbreviations

APC: activated protein C; DrotAA: drotrecogin alfa (activated); ECG: electrocardiogram; EF: ejection fraction; ICU: intensive care unit; INR: international normalized ratio; MAP: mean arterial pressure; PTT: partial thromboplastin time; STEMI: ST-elevation myocardial infarction; TNK: tenecteplase

## Consent

Written informed consent was obtained from the patient for publication of this case report and any accompanying images. A copy of the written consent is available for review by the Editor-in-Chief of this journal.

## Competing interests

The authors declare that they have no competing interests.

## Authors' contributions

LB collected and analyzed all patient data, conducted a literature review and was a major contributor in writing the manuscript. JS collected and analyzed data related to the patient's stay in the intensive care unit. JGP collected, interpreted and analyzed data related to cardiac investigation. RK analyzed all data pertinent to the case, conducted a literature review and was a major contributor in writing the manuscript. All authors read and approved the final manuscript.
